# Sexual Behaviors and Reproductive Responsibility Among Young Black Men at an HBCU: A Qualitative Study

**DOI:** 10.3390/bs16081371

**Published:** 2026-08-10

**Authors:** Zahra Fazli Khalaf, Justin Green, Amanda Lee, Kyla Guilford

**Affiliations:** Psychology Department, College of Health and Human Sciences, North Carolina Agricultural and Technical State University, Greensboro, NC 27411, USA; jrgreen3@aggies.ncat.edu (J.G.); avlee1@aggies.ncat.edu (A.L.); kguilford@aggies.ncat.edu (K.G.)

**Keywords:** black college men, sexual health, reproductive responsibility, masculinity, STI prevention, condom use, pregnancy prevention, qualitative research, higher education

## Abstract

Sexual and reproductive health research has often centered women’s contraceptive and pregnancy-related experiences, while young men’s perspectives on sexual behavior, STI prevention, and reproductive responsibility remain less visible. This qualitative study explored how young Black heterosexual college men understood sexual health knowledge, masculinity, STI risk, condom use, pregnancy prevention, and readiness for fatherhood. Thirteen self-identified Black college men aged 19–22 years participated in individual semi-structured interviews at a Historically Black College/University (HBCU) in the southern United States. Interviews were conducted online, transcribed, de-identified, and analyzed using reflexive thematic analysis. Four themes were identified: fragmented sexual health knowledge and informal learning; learning masculinity through sexual socialization and developing sexual autonomy; experiential learning, STI stigma, and the negotiation of sexual risk; and reproductive responsibility and pregnancy prevention. Participants described limited formal sexual health education, peer and media influences on masculinity, risk judgments shaped by trust and familiarity, and stronger concern for pregnancy prevention than for STI prevention. Findings suggest that sexual health education and campus-based prevention should engage young Black men as reproductive actors with distinct needs, responsibilities, and relational contexts. Programs should integrate STI prevention, condom counseling, partner communication, alcohol-related risk reduction, and reproductive-intention counseling into male-centered, stigma-sensitive approaches.

## 1. Introduction

Sexual and reproductive health encompasses physical, emotional, mental, and social well-being in relation to sexuality and reproduction (World Health Organization, 2010). The college years may involve increased independence, changing relationship contexts, and greater exposure to peer norms, all of which may shape sexual and reproductive health decision-making. In the United States, STIs remain a substantial public health concern, particularly among adolescents and young adults, and surveillance data document pronounced disparities in reported STI rates among Black populations (Centers for Disease Control and Prevention, 2024, 2025). These disparities should not be interpreted as inherent biological or behavioral characteristics of race. Among young Black men, barriers to sexual and reproductive healthcare include limited insurance and affordable access, gaps in knowledge about available services, STI-related stigma, confidentiality concerns, distrust of healthcare providers, and masculine norms that may conflict with care-seeking (Burns et al., 2021).

Young Black men occupy an important but understudied position in sexual and reproductive health research. Existing literature has not provided a comprehensive understanding of how young Black men access and use preventive sexual and reproductive healthcare, particularly STI and HIV testing services (Burns et al., 2021). Research involving college men also indicates that they may endorse shared sexual health responsibility while drawing on gendered expectations that produce uncertainty or inequality in partnered prevention practices (Stanley et al., 2018). However, less is known about how young Black college men understand their own sexual health needs, STI risk, condom use, testing, pregnancy prevention, partner communication, and readiness for fatherhood. Addressing this gap requires attention to how sexual and reproductive decisions are understood and negotiated within interpersonal, social, and institutional contexts (Burns et al., 2021; Stanley et al., 2018). Masculinity is central to this issue because sexual behavior is shaped not only by formal information but also by peer expectations, cultural and media messages, family and religious guidance, and experiences within relationships (Vandello et al., 2024). Precarious manhood theory conceptualizes manhood as a social status that must be earned, publicly demonstrated, and defended; within sexual contexts, heterosexual activity and sexual experience may function as demonstrations of masculinity (Vandello et al., 2024). For Black men, these gendered expectations intersect with racialized constructions that have portrayed Black masculinity as hypermasculine defined by sexual prowess (Lemelle, 2010). Such constructions may influence sexual communication, condom use, testing, and other prevention practices while creating pressure to appear sexually knowledgeable or experienced (Bowleg et al., 2015; Hall & Applewhite, 2013; Vandello et al., 2024). These constructions should be understood as racialized social narratives rather than as inherent characteristics of Black men or Black culture.

At the same time, masculinity is not a fixed or singular determinant of behavior. Research with Black heterosexual men has identified alternative constructions of masculinity centered on taking responsibility for safer sex and maintaining sexual exclusivity, complicating interpretations that frame masculinity only as a source of sexual risk (Bowleg et al., 2015). College men may endorse shared sexual health responsibility while also drawing on gendered assumptions that position women as the primary managers of contraception and pregnancy prevention (James-Hawkins et al., 2019; Stanley et al., 2018). These tensions demonstrate that shared reproductive responsibility may be endorsed as an ideal without being enacted consistently. Condom use may be described as responsible but become inconsistent when men rely on relationship trust, assumptions about partners’ safety, or women’s management of contraception and sexual health risks (Bowleg et al., 2015; Dalessandro et al., 2019; Fazli Khalaf et al., 2026). Sexual health knowledge alone is also insufficient to explain behavior. Students may know that STIs and pregnancy are possible while lacking practical knowledge about symptoms, asymptomatic transmission, testing intervals, partner notification, condom negotiation, or the limits of withdrawal and emergency contraception. University students’ sexual health knowledge may coexist with incomplete prevention practices and assumptions about partner safety (Jahanfar & Fazli Khalaf, 2022). In this context, informal learning from peers, internet searches, social media, partners, pornography, and lived experience can fill gaps left by formal education, but these sources may also reinforce misinformation or stigma.

Existing research indicates that young men’s sexual and reproductive health decisions are shaped by masculine norms, relationship contexts, healthcare knowledge and access, and gendered expectations regarding contraception and sexual health responsibility (Burns et al., 2021; Dalessandro et al., 2019; James-Hawkins et al., 2019; Stanley et al., 2018). However, the literature has given limited attention to how young Black heterosexual college men understand these influences together within an HBCU context. Research on young Black men’s sexual and reproductive health has often emphasized engagement with STI and HIV prevention, testing, and healthcare services, leaving other dimensions of their reproductive decision-making less fully explored (Burns et al., 2021). Research involving college men has also examined how gender norms shape partnered sexual health responsibility and preventive behavior (Stanley et al., 2018). However, less is known about how young Black heterosexual college men connect sexual health learning, risk negotiation, condom use, partner communication, reproductive intentions, and readiness for fatherhood within their own decision-making.

Accordingly, this exploratory qualitative study aimed to examine sexual behaviors and reproductive responsibility among young Black men at an HBCU. Specifically, it explored how participants described sexual health knowledge, masculinity, protective measures, and readiness for fatherhood. The study was guided by one research question: How do young Black men at an HBCU understand and describe their sexual behaviors and reproductive responsibility? Because the study was exploratory and qualitative, no hypotheses were proposed.

## 2. Materials and Methods

### 2.1. Study Design and Setting

This study used a qualitative descriptive design to examine young Black college men’s experiences of sexual behavior, STI prevention, masculinity, and reproductive responsibility. Qualitative description is appropriate when the aim is to provide a comprehensive, data-grounded account of participants’ perspectives and experiences while remaining relatively close to their language and the meanings evident in the data (Sandelowski, 2000, 2010). Although qualitative description involves interpretation, it generally seeks a less abstract account than methodologies oriented toward theory development or deep phenomenological interpretation. This approach was selected because the study sought to understand how participants made sense of sexual and reproductive health in everyday contexts, including peer relationships, campus culture, intimate relationships, and personal decision-making.

The study was conducted at a historically Black university in the southern United States. The HBCU setting was not treated solely as a recruitment site, but as a meaningful social and institutional context shaped by a mission of educating and supporting Black students, culturally affirming campus experiences, peer and gender norms, and access to campus-based resources. These features may influence how Black men understand masculinity, relationships, sexual decision-making, reproductive responsibility, and help-seeking. Accordingly, the HBCU context provided an important setting for examining how racial identity, gender expectations, sexual health, and institutional support intersect in participants’ experiences.

### 2.2. Participants and Recruitment

Purposive sampling was used to recruit participants who met characteristics relevant to the study aim and could provide information about the phenomenon under investigation (Campbell et al., 2020). Eligibility criteria were: self-identification as Black or African American, male, heterosexual, aged 18–24 years, and currently enrolled in or recently attending college. 

Recruitment occurred between September 2025 and February 2026 through social media announcements, student organizations, and snowball referrals. Given the sensitivity of sexual and reproductive health topics and documented barriers to young Black men’s engagement with sexual and reproductive health services, recruitment materials emphasized confidentiality, voluntariness, and respectful participation (Burns et al., 2021). Participants and student contacts were also encouraged to share the study announcement with potentially eligible peers. This referral approach supported recruitment through existing social networks, consistent with snowball sampling methods (Noy, 2008). Study announcements clearly stated the inclusion criteria and were distributed through university-affiliated social media pages and student organizations. Because recruitment occurred through broadly circulated announcements, the number of students who viewed or received the study information could not be determined. Students who expressed interest contacted the research team and confirmed that they met the eligibility criteria before an interview was scheduled. A total of 16 students contacted the research team, and 13 completed interviews. Participants received $20 in compensation for completing the interview. Participants’ education classifications ranged from freshman to senior, and all participants self-identified as heterosexual, African American/Black man, aged 18–22 years (Table 1).

### 2.3. Sample Adequacy

Sample adequacy was evaluated using the concept of information power rather than a predetermined numerical threshold. According to this model, the number of participants needed depends on the focus of the study aim, sample specificity, use of established theory, quality of the interview dialogue, and analysis strategy (Malterud et al., 2016). The sample was highly specific to the research question, as all 13 participants identified as Black heterosexual college men and discussed sexual and reproductive health decision-making within the college context. The focused study aim and semi-structured interviews, which lasted approximately 30–40 min, generated detailed perspectives of sexual health learning, masculine expectations, sexually transmitted infection risk negotiation, condom use, protective practices, and pregnancy prevention. Participants provided both shared and differing perspectives across these areas, allowing the research team to identify and interpret meaningful patterns while retaining variation within the dataset. On the basis of the focused aim, sample specificity, quality and detail of the interviews, and analytical richness of the data, the research team determined that the sample provided adequate information power for the study.

### 2.4. Data Collection

Data were collected through individual semi-structured interviews conducted online using Zoom through institutionally supported accounts. Interviews lasted approximately 30–40 min. Before each interview, participants reviewed the study information, provided informed consent, and were reminded that participation was voluntary and that they could decline to answer any question or withdraw at any time. Participants joined from private locations when possible. Zoom’s automated transcription function was used to generate preliminary transcripts. Members of the research team reviewed the transcripts against the interview recordings, corrected transcription errors, and cleaned and formatted the transcripts before analysis. The semi-structured interview guide was designed to ensure consistent coverage of the core topics while allowing interviewers to use probes and response-based follow-up questions to explore participants’ experiences in greater depth (Kallio et al., 2016). Questions addressed sexual health education, informal information sources, peer and family influences, masculinity, relationships, condom use, STI testing, substance use, pregnancy prevention, fatherhood readiness, and reproductive responsibility. With permission, interviews were audio-recorded, transcribed verbatim, reviewed for accuracy, and de-identified. Participants were assigned participant numbers to protect confidentiality. Files were stored in password-protected institutional systems accessible only to authorized members of the research team.

### 2.5. Data Analysis

Data were analyzed using reflexive thematic analysis following Braun and Clarke’s (2006, 2022) six phases: familiarization with the data, coding, generating initial themes, developing and reviewing themes, refining, defining, and naming themes, and producing the report. Reflexive thematic analysis was used within the qualitative descriptive design to identify and organize patterns across participants’ experiences while remaining close to their language and the meanings expressed in the data. Although the analysis was primarily semantic and descriptive, the research team recognized that coding and theme development were active, interpretive processes shaped by the researchers’ perspectives, reflexivity, and engagement with the data.

Team members first read the transcripts repeatedly to become familiar with the content and context of each interview. Coding was primarily inductive, with initial codes developed from participants’ statements, rather than from a predetermined coding framework. However, the study aims and interview questions served as broad sensitizing guides by directing attention to sexual and reproductive health knowledge, masculinity, sexual decision-making, risk, and responsibility.

Multiple research team members participated in coding and theme development. Team members compared and discussed codes to support reflexive dialogue, examine alternative interpretations, and deepen engagement with the data rather than to establish coder agreement or create a fixed consensus codebook. Differences in interpretation prompted the team to revisit the transcripts, reflect on assumptions, and refine the developing analysis.

Codes were then examined and organized into broader patterns of shared meaning. Candidate themes were reviewed in relation to the coded excerpts and the complete dataset and were refined to preserve both common patterns and variation across participants’ statements. The final analysis focused on four themes: Fragmented Sexual Health Knowledge and Informal Learning; Learning Masculinity Through Sexual Socialization and Developing Sexual Autonomy; Experiential Learning, STI Stigma, and the Negotiation of Sexual Risk; and Reproductive Responsibility and Pregnancy Prevention (see Supplementary Materials). Ellipses in quotations indicate that separated portions of the same response were joined without changing meaning. 

### 2.6. Trustworthiness and Reflexivity

Trustworthiness was supported through attention to credibility, dependability, confirmability, and transferability (Lincoln & Guba, 1985; Nowell et al., 2017). All interviewers were trained student researchers who participated in role-play interviews and discussed consistent interviewing procedures before data collection. Interviewers were not enrolled in the same classes as participants and did not belong to their immediate social circles. This arrangement was intended to reduce the influence of preexisting relationships, perceived pressure, power imbalances, and concerns about confidentiality. Participants were also asked whether they preferred a male or female interviewer, and their preferences were accommodated when possible.

All interviewers identified as Black, while the data analysis team also included a non-Black researcher. The research team recognized that members’ racial identities, gender, professional roles, and experiences could shape interactions with participants and interpretations of the data. During reflexive discussions, team members considered whether interviewer identities and experiences, including the perspectives and field notes of the male interviewer, influenced data collection or interpretation. Differences in interpretation were discussed and examined against the transcripts and interview notes. Although the team did not identify major concerns arising from interviewer differences, reflexive discussion continued throughout the analytic process. Multiple team members contributed to coding, analytic discussion, and interpretation. These discussions were used to examine assumptions, consider alternative interpretations, and strengthen engagement with the data rather than to establish coder agreement. Analytic decisions, coding changes, and theme development were documented through research notes and an audit trail.

## 3. Results

Four themes were identified: fragmented sexual health education and reliance on informal learning; masculine sexual socialization alongside growing autonomy; relational and experiential negotiation of STI risk; and a strong emphasis on pregnancy prevention and readiness for fatherhood. The themes are presented below with attention to how participants moved between knowledge, social expectation, relationship context, and personal responsibility when making sexual and reproductive health decisions. Table 2 shows a summary of findings.

### 3.1. Fragmented Sexual Health Knowledge and Informal Learning

Participants did not describe sexual health knowledge as a single body of information received from one trusted source. Instead, they recalled learning in pieces: in middle or high school, through campus programs, from parents, through peers, from social media, and through their own experiences. Formal education often introduced broad topics such as anatomy, puberty, pregnancy, abstinence, condoms, or sexually transmitted infections, but many participants felt that it did not prepare them for the practical questions that emerged later in relationships and college life.

For some, school-based education was present and memorable, but still limited in scope. Participant 11 described receiving a limited foundation:

“In terms of actually talking about sex, I don’t really think I got that in school… I knew broadly what sex was, but not much detail… As I got older, I researched and talked to people… I was basically given the bare minimum growing up.”

Participant 1 similarly distinguished between being told that STIs existed and understanding their meaning:

“I could be more informed about STIs and what they really are… They kind of just tell you, oh, you can get this and that, but not what this and that really are…”

Several participants contrasted general awareness with deeper comprehension. Participant 2 wanted more information about the consequences of untreated infections and available protection methods:

“I guess what happens if you don’t get treated? I forgot how that process works… also the different types of protection… I know there’s condoms and things of that sort, but… there’s other forms of protection out there.”

This distinction between exposure and understanding was important. Participants were rarely saying that they had never heard of STIs, condoms, or pregnancy. Rather, they described gaps in applied knowledge: how to recognize symptoms, what to do after exposure or diagnosis, how to speak with partners, how to seek care without embarrassment, and how to evaluate information found online or through peers. Participant 5 summarized this practical gap simply:

“What to do once you get an STI or STD?”

When formal instruction was absent or inadequate, participants filled the space through informal learning. Peer conversations normalized sexual topics and offered social cues, but they were not always reliable or complete. Participant 9 explained:

“I don’t believe I did sex ed in high school… It was just kind of like you just… got to a certain age, and, you know, that’s what your classmates spoke about, and you eventually developed an understanding of it.”

Family members also shaped what participants considered important. Some parents focused strongly on avoiding early pregnancy, while others framed sex through religious or moral expectations. Online sources and social media were used to answer questions, yet participants were aware that these sources varied in quality. Participant 1 noted the usefulness and uncertainty of social media information:

“A lot of the sources are from, like, school, but also, like, any stuff that you’ll see on social media and things like that, that are helpful. I want to know if they’re the most reliable, because it’s from social media, but they are helpful.”

Campus-based resources also contributed to participants’ sexual health learning, although engagement varied. Some participants reported directly using campus health services, particularly STI testing. Participant 12 described returning to the campus health center for testing after sexual partners:

“The campus health center… About three times after partners… I get tested.”

Participants also described exposure to freshman-level sexual health programs that addressed topics such as safer sex, STIs, and protection. Others were aware that the university provided free testing, condoms, health-center services, and educational programs but did not clearly indicate that they had personally used them. Participant 8 emphasized that access depended partly on students taking the initiative: “The resources are there, but you have to seek them out.”

Thus, campus resources were available and used by some participants, but awareness and engagement were uneven. The findings suggest that the presence of services alone did not ensure that students would access them or receive the practical information they needed. 

Viewed together, this theme shows sexual health learning as cumulative, uneven, and highly context dependent. Participants entered college with some knowledge, but they continued to assemble understanding through campus exposure, partner experiences, media, family messages, and trial-and-error learning. This fragmented pathway created room for both caution and misinformation: participants could recognize that sexual behavior carried risk while still feeling underprepared to manage prevention, testing, treatment, and partner communication in concrete situations.

### 3.2. Learning Masculinity Through Sexual Socialization and Developing Sexual Autonomy

Participants described masculinity as shaped through observation, peer conversations, media, family expectations, and exposure to broader racialized narratives about Black male sexuality. Their experiences reflected social expectations that young men should demonstrate sexual experience, heterosexual desirability, or social confidence. These narratives are presented here as racialized social constructions that participants encountered and negotiated, rather than as accurate descriptions of Black men or Black culture.

Participant 1 described pressure to be perceived as sexually active:

“As a male, it’s always like… you’re supposed to be active… it’s a type of peer pressure, because you want to be kind of seen as… I’m sexually active.”

Participant 2 used the terms “Black culture” and “hypersexual” when describing how he perceived messages circulating among peers and in society:

“Society, and particularly with my friends, were… in kind of… a sex addict culture… and I’d also just say… Black culture as well… as far as being hypersexual.”

This language reflected the participant’s interpretation of a racialized sexual narrative rather than an endorsement or characterization of Black culture by the research team. His description illustrates how stereotypes of Black male hypersexuality may become part of the social environment through which some young men interpret expectations surrounding sexual behavior.

Participants described encountering sexual expectations before college and through campus life, peer interactions, and digital media. Participant 7 explained how peers, media, and claims of sexual experience initially shaped his curiosity:

“My peers and I discussed things we liked, and that influenced me… Social media was my first introduction and sparked curiosity… In high school, popularity and claims of sexual experience influenced my curiosity.”

Media also contributed to cultural scripts concerning when young men were expected to become sexually active. Participant 10 described portrayals of sex as a marker of age or maturity:

“Society makes it seem like you’re supposed to have sex by a certain age… A lot of teen movies focus on someone losing their virginity… a character feeling pressure to have sex just because of his age.”

Participants did not uniformly accept or reproduce these messages. Many described developing greater autonomy, self-control, and confidence in making decisions based on personal readiness. Some rejected peer pressure, while others explained that it had become less influential as they matured. Participant 9 explicitly challenged the racialized and gendered notion that sexual experience establishes manhood:

“Especially the stigma with men, and African American men especially… you don’t truly become a man once you’ve had sex… And with me, it’s no major rush.”

Participant 12 similarly framed sexual activity as an individual decision rather than a requirement for social acceptance:

“Sex isn’t based on what others do… It’s weird to copy others… It should be something you want.”

Resistance to sexual expectations was also connected to religion, family values, and personal readiness. Participant 10 described tension between sexual desire and his religious commitment:

“I’m Christian, and the Bible teaches waiting until marriage… It’s a goal in part of my mind, even though another part of me struggles with it.”

Participants also described peer environments that challenged, rather than reinforced, expectations of sexual performance. Participant 8 explained:

“My close friend group doesn’t glorify sleeping with a lot of people. There’s more of a positive circle, and promiscuity isn’t really encouraged.”

This theme therefore reflects participants’ negotiation of racialized and gendered sexual narratives rather than the validation of stereotypes about Black male sexuality. Although some participants encountered expectations that men should appear sexually experienced, their descriptions also emphasized resistance, restraint, religious and moral reflection, care for partners, personal readiness, protection, and future planning. These narratives demonstrate how participants developed sexual autonomy while defining masculinity on their own terms.

### 3.3. Experiential Learning, STI Stigma, and the Negotiation of Sexual Risk

Participants generally understood that sex could involve STI exposure, pregnancy, emotional consequences, and relationship complications. However, risk was not assessed only through formal knowledge. In practice, participants evaluated risk through the perceived trustworthiness of a partner, the length or seriousness of a relationship, whether a partner seemed honest, whether testing had been discussed, whether condoms were available, and whether alcohol or emotional vulnerability was involved. Prevention was therefore relational and situational, not simply informational.

Participant 10 located risk in uncertainty about partners:

“You don’t know how many people someone may be involved with… I know what I’m doing, but I don’t know what they’re doing, and anything could happen.”

Participant 4 similarly emphasized that risk could remain even when someone appeared safe or trustworthy:

“There always is… some people do lie… sometimes people don’t even know they have it… So, you just gotta be real careful.”

Some participants described protective strategies that combined testing, condoms, and partner discussion. Participant 2 framed testing as something that could occur with a partner before intercourse:

“Going to the health clinic with your partner and checking each other’s statuses before you indulge in sexual intercourse.”

Yet the same interviews also revealed the limits of these strategies. Participants sometimes relied on visual impressions, familiarity, or trust instead of current STI testing. Participant 2 combined questions about testing with subjective judgments:

“I say, have you been tested?… I just kind of look at the person… I kinda trust that whoever I do choose… They’re, clean… STD-free… But, you know, that’s not always the case.”

A central insight across participants’ statements was that condom use was often understood more strongly in relation to pregnancy prevention than STI prevention. This distinction became particularly visible when the absence of pregnancy was interpreted as evidence that condom use was no longer necessary. Participant 13 described discontinuing condoms within an ongoing relationship:

“At first, yeah, I would use condoms. And then it got to a point where we just wouldn’t use them anymore… because we would see that she wouldn’t get pregnant.”

The absence of pregnancy appeared to function as a misleading indicator of safety, even though it provided no information about STI exposure and did not eliminate the possibility of a future pregnancy. This account illustrates how pregnancy-focused interpretations of condoms could weaken dual-protection practices.

Participant 1 also described knowingly continuing sexual activity after recognizing potential risk:

“Has there been times when you recognized a risk, but chose to engage in sexual activity anyway?… Yes… a circumstance would be, like, taking the condom off… even if it was someone I didn’t know that well.”

Regardless of whether the condom removal was consensual, the statement reflects a decision to proceed without barrier protection despite uncertainty about the partner and possible STI exposure. More broadly, these examples illustrate how participants sometimes recognized sexual risk but made situational decisions shaped by relationship familiarity, perceptions of pregnancy risk, attraction, convenience, or the circumstances of the encounter.

Alcohol also influenced sexual decision-making for some participants. Participant 8 explained:

“Drinking has led to situations I probably wouldn’t have been in if I were sober… ending up back at my apartment, and having sex.”

Participant 9 similarly linked intoxication with reduced capacity for careful decision-making:

“It’s very unsafe, because you’re not in your right mind… things may happen… you’re not in the right mind to make those decisions.”

Emotional states also influenced sexual desire and decision-making, although participants described different responses. Participant 1 explicitly described using sexual activity to cope with boredom and emotional pain following relationship betrayal:

“When you’re in a more vulnerable or bored state of mind, you’re more likely to do more sexual acts… when I got cheated on, I was using sexual activity because I was bored and didn’t understand my feelings… it was something I was doing as a coping mechanism, and it didn’t really help at all.”

For other participants, mood and stress affected sexual desire without necessarily leading to sex as a coping response. Participant 6 explained:

“If I’m feeling good, I’m more likely to want sex. If I’m down, less likely. Stress can increase my desire.”

Participant 7 similarly described being influenced by emotions while attempting to regulate his responses:

“It does influence me, but I try to control my emotions before acting… Finding balance is something I’m actively working on.”

Emotional distress could also reduce sexual interest. Participant 2 stated:

“Lately, my emotional state hasn’t been the best… when I’m depressed or not feeling up to par… I just don’t have sex.”

These experiences indicate that emotional well-being shaped sexual interest and decision-making for several participants, but the direction of this influence varied. Using sexual activity specifically as a coping mechanism was described most clearly by Participant 1 and is therefore not interpreted as a pattern across the entire sample.

For participants who had experienced an STI, risk became embodied rather than theoretical. Participant 6 described both emotional and behavioral consequences:

“I experienced an STI… I felt angry and didn’t want to have sex afterward… I got treated and was fine after 14 days… I withdrew socially but still attended classes.”

Participant 1 also described the fear that followed testing and diagnosis:

“I was feeling sick for a little bit… I went and got tested at the health center… in the moment, I thought it was worse than it really was… the afterthought is always worse than the moment, because you think, why did I do that? What did I actually gain?… then usually I would get tested after.”

This theme shows that sexual risk negotiation was shaped by more than knowledge alone. Participants’ decisions were influenced by partner trust, relationship familiarity, STI stigma, alcohol use, emotional states, and the circumstances of the encounter. A central finding was that condoms were sometimes understood primarily as a means of preventing pregnancy, allowing the absence of pregnancy to become misleading evidence of safety despite continued STI risk. Participants also described recognizing risk while making situational decisions that did not consistently align with their stated intentions. At the same time, experiences with testing, diagnosis, treatment, and reflection created opportunities for greater awareness and changes in sexual decision-making.

### 3.4. Reproductive Responsibility and Pregnancy Prevention

Pregnancy prevention was one of the most consistent expressions of responsibility in the interviews. Participants often spoke about pregnancy as more life-changing, immediate, and socially visible than STI risk. They connected pregnancy to education, finances, readiness for fatherhood, partner well-being, and family expectations. Although condom use was not always consistent for STI prevention, many participants described pregnancy avoidance as central to being responsible.

Participant 4 described pregnancy as a shared consequence:

“Pregnancy… doesn’t just affect the man, it affects the other person as well… you just have to make sure that you’re ready for that moment.”

Participant 7 connected prevention with preparation for fatherhood:

“My family taught me to be cautious and aware that sex can lead to a child… It motivates me to be prepared to support a future child.”

Family messages about timing were especially strong. Some participants had been taught that early parenthood could alter education and economic stability. Participant 9’s perspective was shaped by his parents’ experience of early parenthood:

“My parents had my sister as teenagers… safe sex is a big thing that my parents really preach… having that child so early… really made it difficult for them.”

Participant 12 expressed a similar family message:

“My family says don’t have kids young… That affects my decisions… They would support me.”

Pregnancy prevention was also linked to participants’ own educational and developmental timelines. Avoiding fatherhood was not framed only as avoiding responsibility; it was often described as postponing fatherhood until they could provide materially and emotionally. Participant 6 summarized the timing expectation clearly:

“No kids before 25.”

Participant 12 primarily explained condom use in relation to preventing pregnancy:

“I would prioritize using a condom… It lessens the chance you’ll have a kid… I try to avoid pregnancy.”

Several participants also described responsibility in relational terms. Pregnancy was not imagined as affecting only the man’s future; it was understood as involving a partner’s body, options, well-being, and future. Participant 9 linked safety to care for both people involved:

“Having a child, or contracting something can be world-changing… you should, first and foremost, care for not only yourself, but the other person… you should definitely try to be as safe as possible.”

At the same time, responsibility did not always translate into comprehensive prevention. Some participants described condoms, withdrawal, Plan B, testing, or partner communication, but these strategies were not always applied consistently or did not protect equally against both pregnancy and STIs. Participant 1 made this inconsistency visible:

“With multiple partners, I try to make sure I get tested after every partner. With pregnancy, I try to use condoms, but there are times where I don’t… I pull out or use Plan Bs… that’s how I avoid risk, but there are times where I’m not the best with avoiding risk.”

This final theme shows that young Black college men in the study were not indifferent to reproductive responsibility. Many expressed clear concern about avoiding unintended pregnancy, delaying fatherhood, and protecting partners. The tension was between values and practice: participants often articulated responsibility more strongly than they described consistent, dual-protection behavior. Their narratives suggest that male-centered sexual health education should build on this existing sense of responsibility while addressing the practical gap between intention, communication, contraception, and STI prevention.

## 4. Discussion

This study examined how young Black college men understood sexual behavior, STI risk, masculinity, and reproductive responsibility. The findings indicate that sexual and reproductive health decision-making was shaped by incomplete formal education, informal learning networks, masculine sexual socialization, relationship-based trust, alcohol-related circumstances, STI stigma, and concerns about readiness for fatherhood. Taken together, these findings support understanding young Black men not merely in relation to reproductive risk, but as sexual and reproductive decision-makers who negotiate responsibility, uncertainty, care, and social pressure within specific relational and institutional contexts.

The first theme showed that participants often received fragmented sexual health information. Formal education introduced topics such as puberty, pregnancy, abstinence, and STIs, but did not consistently provide the practical knowledge participants needed to apply prevention strategies in everyday situations. This finding is consistent with the World Health Organization’s emphasis on comprehensive sexual health programming that addresses education, social and cultural context, access to information, and appropriate health services (World Health Organization, 2010). Participants described filling educational gaps through peers, internet searches, social media, partners, and personal experience. Although these informal sources made sexual health information more accessible, participants’ uncertainty about their reliability suggests that such sources may also contribute to incomplete or inaccurate understandings of STI transmission, treatment, and pregnancy prevention.

The second theme highlights the importance of masculinity in sexual socialization. Participants described pressure to be sexually active, appear experienced, and connect sexual behavior with manhood or social standing. This finding aligns with precarious manhood research suggesting that heterosexual activity and sexual experience may function as ways of establishing or demonstrating masculinity during young men’s sexual development (Vandello et al., 2024). However, the findings complicate the assumption that masculine norms inevitably produce sexual risk. Several participants rejected the idea that sexual experience defines manhood and instead emphasized readiness, personal judgment, faith, comfort, or relational commitment. These findings suggest that behavioral interventions could build on participants’ existing values of autonomy, care, and responsibility rather than framing masculinity only as a risk factor.

The third theme showed that STI risk was negotiated within relationships. Participants recognized that partner history and condomless sex could involve risk, but trust, familiarity, presumed honesty, and perceptions of partner safety often shaped their protection decisions. This finding is consistent with research showing that college men’s contraceptive and sexual-health decisions are influenced by gendered expectations, relationship dynamics, and assumptions about partnered responsibility (Dalessandro et al., 2019; Stanley et al., 2018).

In practice, participants sometimes treated emotional trust, familiarity, or presumed honesty as substitutes for current testing, consistent condom use, or explicit communication about STI exposure. Prevention programs should therefore help students distinguish trust as a relational value from verified STI status as a health practice. This distinction can be addressed without stigmatizing relationships or discouraging intimacy.

Alcohol-related circumstances further complicated decision-making. Participants’ descriptions suggested that alcohol did not independently determine sexual risk but could create situations in which communication, planning, and protection became less consistent. Campus prevention efforts could therefore supplement general warnings about alcohol and sex with anticipatory strategies, including carrying condoms, discussing boundaries and protection before drinking, recognizing situations in which decision-making may be impaired, and seeking appropriate testing or care following condomless sex.

STI stigma also emerged as an important concern. Participants’ use of the term “clean” to describe presumed STI-negative partners may reinforce a moral distinction between people with and without an STI and make communication, testing, disclosure, or care-seeking more difficult. One participant described anger, social withdrawal, and temporary avoidance of sex following an STI diagnosis, illustrating that diagnosis may have emotional and social consequences in addition to requiring medical treatment. This interpretation is consistent with research identifying STI-related stigma, mistrust, gaps in knowledge about services, and masculine expectations as barriers to sexual and reproductive healthcare among young Black men (Burns et al., 2021). Stigma-sensitive education could normalize testing as a routine health practice, explain asymptomatic infection, discourage moralizing language, and provide practical guidance regarding disclosure, partner notification, treatment, and prevention of reinfection.

The fourth theme showed that participants expressed a stronger sense of responsibility for pregnancy prevention than for STI prevention. They connected pregnancy avoidance with educational goals, financial readiness, partner well-being, and preparation for future fatherhood. This finding both supports and extends previous research. College men may endorse equal contraceptive responsibility while remaining silent during decision-making or deferring contraceptive management to women (Dalessandro et al., 2019; James-Hawkins et al., 2019). In the present study, participants articulated responsibility for preventing pregnancy, yet some descriptions indicated that pregnancy prevention was prioritized over STI prevention. This distinction is important because practices used to reduce pregnancy risk do not necessarily protect against STIs. Counseling and education should therefore address pregnancy and STI prevention together while recognizing that young men may interpret these risks differently. Greater attention to male contraceptive options and male-centered reproductive healthcare may promote shared responsibility and reduce the assumption that women should bear the primary burden of pregnancy prevention (Kidula et al., 2025).

This study contributes specific insight into how young Black heterosexual college men at an HBCU understood and negotiated sexual and reproductive responsibility. Participants did not simply reproduce racialized and gendered expectations about masculinity; many questioned sexual performance as a marker of manhood and described autonomy through readiness, restraint, faith, care, and future planning. Pregnancy prevention was often more salient than STI prevention and was closely connected to educational goals, financial preparation, partner well-being, and readiness for fatherhood. At the same time, the absence of pregnancy could become misleading evidence of safety and contribute to condom discontinuation despite continued STI risk. Participants’ decisions were also shaped by relational trust and situational judgment, demonstrating that awareness of risk did not necessarily result in consistent protective practices. Finally, the HBCU context included educational programs, testing, condoms, and health services, but awareness and use of these resources varied. Together, these findings extend existing research by showing how masculinity, reproductive intentions, relationship dynamics, and institutional context intersect in young Black college men’s sexual and reproductive decision-making.

The findings support a three-part framework for campus-based sexual and reproductive health programming: practical knowledge, relational decision-making, and accessible support. Service providers could implement the practical-knowledge component through brief workshops, orientation sessions, digital modules, and individual counseling that distinguish STI prevention from pregnancy prevention and address asymptomatic infection, testing, condom use, emergency contraception, and reproductive intentions. These activities should use scenarios that reflect common decisions described by participants, such as discontinuing condoms in trusted relationships or interpreting the absence of pregnancy as evidence of safety. The relational component could be incorporated into counseling, peer education, and campus outreach by helping students practice communication about STI testing, condom use, contraception, consent, exclusivity, alcohol-related situations, and readiness for parenthood. Rather than presenting masculinity only as a risk factor, providers should affirm autonomy, preparation, honesty, care for partners, and mutual responsibility. Campus health centers could support the accessible-care component by offering confidential testing, readily available condoms, flexible appointment options, and clear information about where and how to obtain services. Partnerships with student organizations, residence-life staff, counseling services, and trained peer educators may help reach students who do not independently seek services. Programs should also be evaluated through service-use data, anonymous student feedback, and assessments of changes in knowledge, communication confidence, and preventive practices. Several limitations should be considered. The sample was small, college-based, and drawn from a single HBCU in the southern United States; therefore, the findings are context-specific and are not statistically generalizable to all Black men, HBCU students, or college populations. In addition, the findings may not transfer directly to young Black men who do not attend college. Although some influences may be shared, non-college populations may differ in healthcare access, social networks, and available sexual health resources. Future studies should examine these issues in community and non-college settings. Purposive and snowball sampling may have introduced recruitment, self-selection, and network bias by reaching students who were more socially connected, more comfortable discussing sexual health, or more interested in the topic. Students who did not engage with university organizations or the social media channels through which recruitment materials were distributed may have been less likely to participate.

The study relied on self-reported experiences involving sensitive sexual behaviors, which may have been affected by recall and social-desirability bias. Participants were not asked to review the transcripts, preliminary interpretations, or final themes; therefore, the findings were not verified through participant checking. Although interviewers were trained, participated in role-play, followed common procedures, and were not in the same classes or immediate social circles as participants, interviewer identity, gender, communication style, and perceived similarity to participants may still have influenced disclosure. Researcher identities and experiences may also have shaped interpretation despite ongoing reflexive discussion. Interviews were conducted online, and although participants were encouraged to join from private locations, the research team could not fully ensure privacy or determine whether the possibility of being overheard affected participants’ responses.

The study also included only the perspectives of the young men and did not include partners, whose perspectives could provide additional insight into communication, contraception, consent, trust, and shared reproductive responsibility. Interview length and depth varied, and some topics were explored more fully with certain participants than with others. Future research should include larger multi-campus samples, more detailed demographic information, partner or dyadic perspectives, and longitudinal approaches examining how sexual and reproductive decisions change over time. 

## 5. Conclusions

Young Black college men’s sexual and reproductive health decisions were shaped by fragmented education, masculine sexual socialization, relational trust, STI stigma, alcohol-related circumstances, emotional influences, and lived experience. Many participants rejected the idea that sexual activity defines manhood, yet inconsistent condom use, assumptions about partner safety, and limited practical sexual health knowledge sustained vulnerability to STIs. Pregnancy prevention was more consistently understood as a male responsibility and was linked to educational goals, financial stability, partner well-being, and readiness for fatherhood. However, interpreting condoms primarily through pregnancy prevention could allow the absence of pregnancy to serve as misleading evidence of safety despite continued STI risk. Campus-based interventions should engage young Black men as reproductive actors and provide confidential, culturally responsive, and stigma-sensitive support that integrates practical sexual health knowledge, relational communication, and accessible services.

## Figures and Tables

**Table 1:** Participant characteristics.

Participant	Age (Years)	Education Classification	Race/Ethnicity	Relationship Status
P1	20	Junior	African American/Black	In a relationship
P2	21	Junior	African American/Black	Currently single
P3	20	Junior	African American/Black	In a relationship
P4	21	Junior	African American/Black	Currently single
P5	19	Sophomore	African American/Black	Currently single
P6	20	Junior	African American/Black	Currently single
P7	22	Senior	African American/Black	In a relationship
P8	22	Senior	African American/Black	In a relationship
P9	19	Sophomore	African American/Black	Currently single
P10	20	Sophomore	African American/Black	In a relationship
P11	19	Sophomore	African American/Black	Currently single
P12	19	Freshman	African American/Black	Currently single
P13	21	Junior	African American/Black	In a relationship

Note. “Currently single” indicates that the participant was not in a committed relationship at the time of the interview. This classification does not preclude engagement in casual sexual relationships or one-time sexual encounters.

**Table 2:** Summary of Themes and Key Findings.

Theme	Key Finding
Fragmented Sexual Health Knowledge and Informal Learning	Participants assembled sexual health knowledge from formal education, family, peers, media, campus resources, and personal experience, but practical knowledge gaps remained.
Learning Masculinity Through Sexual Socialization and Developing Sexual Autonomy	Participants encountered racialized and gendered expectations about sexual activity while also developing autonomy through personal values, readiness, restraint, and responsibility.
Experiential Learning, STI Stigma, and the Negotiation of Sexual Risk	Sexual risk decisions were shaped by trust, familiarity, stigma, emotions, alcohol, and experience; condom use was sometimes interpreted more through pregnancy prevention than STI protection.
Reproductive Responsibility and Pregnancy Prevention	Participants emphasized avoiding unintended pregnancy and delaying fatherhood until they were financially and emotionally prepared, although dual-protection practices were inconsistent.

## Data Availability

The qualitative interview data are not publicly available due to the sensitive nature of the topic and participant confidentiality. De-identified data may be available from the corresponding author upon reasonable request and subject to institutional ethical requirements.

## Supplementary Materials

The following supporting information can be downloaded at: https://www.mdpi.com/article/10.3390/bs16081371/s1.
