# Supplementary material for: Sexual Behaviors and Reproductive Responsibility Among Young Black Men at an HBCU: A Qualitative Study
Source: Behav Sci (Basel). 2026 Aug 10;16(8):1371. doi: 10.3390/bs16081371 (PMC13509867; doi:10.3390/bs16081371)
Supplement: Supplementary file 1 [file behavsci-16-01371-s001.zip › behavsci-4436082-supplementary.pdf]

Table S1. Final Analytic Codes

Analytic approach. The codebook reflects the four final themes reported in the Results. Coding was primarily inductive and semantic. The listed items are analytic codes used to organize data within each theme; they are not presented as formal subthemes. Participant quotations are intentionally omitted from this appendix because selected de-identified excerpts are already presented in the Results and the study concerns sensitive sexual and reproductive health experiences.

Final theme	Analytic code	Operational definition	Include when	Exclude or distinguish when
**Fragmented Sexual Health Knowledge and Informal Learning**	Formal sexual health education	Accounts of school- or university-based instruction on sex, anatomy, pregnancy, contraception, condoms, STIs, abstinence, or related topics.	Code when participants describe what was taught, where it was taught, or how formal instruction was delivered.	Do not use for information learned primarily from family, peers, media, partners, or personal experience.
	Limited depth and applied knowledge	Perceived gaps between general awareness of sexual health topics and practical knowledge needed for prevention, testing, treatment, communication, or care seeking.	Code statements about receiving only basic information, not understanding consequences or treatment, or wanting more practical guidance.	Do not use when the participant simply reports having no formal education without describing a knowledge gap.
	Family and peer learning	Sexual health information, norms, warnings, and advice learned through parents, relatives, friends, classmates, or other interpersonal networks.	Code family messages, peer conversations, shared advice, and socially learned understandings.	Do not use when peers are discussed primarily as creating masculinity pressure; use the relevant Theme 2 code.
	Digital and media-based learning	Information or ideas about sexual health obtained through social media, online searches, pornography, television, film, or other digital sources.	Code both useful learning and uncertainty about the accuracy or realism of digital information.	Do not use when media is discussed mainly as prescribing masculinity or the expected timing of sex; use the relevant Theme 2 code.
	Campus resources and student initiative	Awareness and use of campus health centers, testing, condoms, educational programs, and other university sexual health resources.	Code availability, accessibility, visibility, affordability, and the need for students to actively seek resources.	Do not use for personal testing or condom decisions unless the focus is the resource itself.
	Cumulative and experiential learning	Sexual health knowledge assembled over time through multiple partial sources and lived experiences rather than one comprehensive source.	Code descriptions of trial-and-error learning, later self-education, and continued learning after entering college.	Do not use for specific STI experiences when the main focus is risk, stigma, or behavior change; use Theme 3.
**Learning Masculinity Through Sexual Socialization and Developing Sexual Autonomy**	Masculinity as sexual performance	Expectations that men should be sexually active, experienced, assertive, desirable, or able to demonstrate manhood through sex.	Code statements linking sexual activity or experience with maturity, credibility, popularity, or masculinity.	Do not use for general peer influence unrelated to gendered expectations.
	Racialized hypersexual expectations	Perceived stereotypes or cultural narratives portraying Black men as naturally hypersexual or expected to display heightened sexual interest or activity.	Code explicit references to Black male sexuality, hypersexuality, or racialized expectations.	Do not infer racialized meaning when the participant discusses masculinity without mentioning race or Black identity.
	Peer pressure and social comparison	Direct or indirect influence from friends, classmates, popularity, group conversation, or perceived peer norms on sexual curiosity or behavior.	Code pressure, teasing, comparison, assumptions, or efforts to fit in.	Do not use when peers function primarily as sources of sexual health information; use Theme 1.

Final theme	Analytic code	Operational definition	Include when	Exclude or distinguish when
	Media sexual scripts	Media representations that normalize sex, glorify multiple partners, create unrealistic expectations, or suggest that sex should occur by a certain age.	Code media messages about what men should want, do, or have already experienced.	Do not use when the focus is evaluating the reliability of health information found online; use Theme 1.
	Family, cultural, and religious expectations	Moral, relational, cultural, or religious messages that shape how participants interpret sex, abstinence, marriage, readiness, and acceptable behavior.	Code religious teachings, family values, sacredness, traditional norms, and relationship expectations.	Do not use when the primary emphasis is avoiding early pregnancy or preparing for fatherhood; use Theme 4.
	Resistance and developing autonomy	Movement toward making sexual decisions according to personal readiness, values, boundaries, and judgment rather than external pressure.	Code rejection of peer pressure, delaying sex, setting boundaries, self-control, and statements that sex is an individual choice.	Do not use for protective behaviors unless autonomy or resistance is the central meaning.
	Responsibility-oriented masculinity	Understandings of manhood centered on restraint, care for partners, protection, testing, readiness, or future planning rather than sexual conquest.	Code narratives that redefine masculinity through responsibility and thoughtful decision-making.	Do not use for pregnancy-specific responsibility when fatherhood or contraception is central; use Theme 4.
**Experiential Learning, STI Stigma, and the Negotiation of Sexual Risk**	Partner trust and perceived safety	Assessment of sexual risk through familiarity, relationship status, perceived honesty, appearance, or knowledge of a partner's history.	Code reliance on trust, visual impressions, relationship length, or perceived character when judging safety.	Do not use for confirmed testing or explicit preventive practices unless those are the focus.
	Testing and partner communication	Use or discussion of STI testing, status disclosure, partner histories, boundaries, and communication before or after sexual activity.	Code joint testing, asking about status, discussing histories, or communication as a protective strategy.	Do not use for campus testing availability unless the focus is institutional access; use Theme 1.
	Condom use and protective practices	Use of condoms or other practices intended to reduce STI exposure during sexual activity.	Code condom availability, routine use, partner negotiation, or decisions to stop sexual activity when protection is unavailable.	When the primary purpose is pregnancy prevention rather than STI prevention, use Theme 4.
	Recognized risk and inconsistent protection	Situations in which participants understood a possible risk but engaged in sexual activity or discontinued protective practices.	Code condom removal, unprotected sex, sex with uncertain partner history, or acknowledged risk-taking.	Do not use for hypothetical risk perceptions without an actual or contemplated behavioral decision.
	STI stigma and moralized status language	Language or beliefs that attach shame, blame, contamination, or moral judgment to STI status, testing, treatment, or disclosure.	Code terms such as 'clean,' embarrassment, fear of judgment, withdrawal, or reluctance to disclose or seek care.	Do not use for fear of medical consequences without stigma or moral judgment.
	Alcohol, substances, and impaired judgment	Influence of alcohol or other substances on inhibition, consent, partner selection, condom use, or sexual decision-making.	Code intoxication-related encounters, reduced control, impaired judgment, and concerns about consent.	Do not use when a participant only states that substances had no influence unless contrasted with perceived risk.
	Emotional vulnerability and coping	Influence of loneliness, betrayal, boredom, stress, depression, attraction, or other emotional states on sexual desire or behavior.	Code sex used as coping, reduced sexual activity during distress, or reflection on emotional readiness.	Do not use when the main focus is moral or religious readiness; use Theme 2.

Final theme	Analytic code	Operational definition	Include when	Exclude or distinguish when
	STI experience and behavior change	Personal experience with symptoms, testing, diagnosis, treatment, fear, withdrawal, regret, or subsequent changes in behavior.	Code embodied or lived STI experiences and learning that followed them.	Do not use for general knowledge gaps about STI treatment without personal experience; use Theme 1.
	Situational risk negotiation	Real-time balancing of attraction, relationship context, condom availability, partner familiarity, alcohol, emotion, and anticipated consequences.	Code accounts showing that risk decisions changed across situations rather than following a single fixed rule.	Use a more specific Theme 3 code when one factor is clearly the dominant meaning.
**Reproductive Responsibility and Pregnancy Prevention**	Pregnancy as a life-changing consequence	Perceptions of pregnancy as immediate, visible, consequential, or capable of altering education, finances, relationships, or future plans.	Code descriptions of pregnancy as especially serious or world-changing.	Do not use for STI consequences unless pregnancy is explicitly compared with them.
	Shared reproductive responsibility	Recognition that pregnancy affects both partners and that men have responsibilities related to communication, prevention, support, and partner well-being.	Code care for partners, shared consequences, mutual decision-making, and responsibility beyond self-protection.	Do not use for general relationship trust or STI communication; use Theme 3.
	Fatherhood readiness and delayed parenting	Desire to postpone fatherhood until participants feel emotionally, educationally, relationally, or financially prepared.	Code age or timing goals, readiness to provide, and future-oriented planning for fatherhood.	Do not use for broad sexual restraint unless fatherhood or parenting readiness is central.
	Family messages about early pregnancy	Family histories, warnings, or expectations emphasizing the consequences of becoming a parent at a young age.	Code parental advice, family examples of early parenthood, and messages about completing education or becoming stable first.	Do not use for family religious teachings unrelated to pregnancy; use Theme 2.
	Pregnancy prevention practices	Practices intended primarily to prevent pregnancy, including condoms, withdrawal, emergency contraception, partner contraception, and planning.	Code strategies described specifically as avoiding pregnancy or delaying fatherhood.	When condoms are discussed mainly for STI protection, use Theme 3.
	Intention-practice and dual-protection gap	Tension between stated responsibility and inconsistent use of methods that protect against both pregnancy and STIs.	Code strong intentions alongside inconsistent condom use, reliance on withdrawal or emergency contraception, or separate approaches to pregnancy and STI prevention.	Do not use when prevention practices are described as consistent and comprehensive.

Table S2. Semi-Structured Interview Domains Relevant to the Present Analysis

Note. Interviews began with rapport building, confirmation of informed consent and confidentiality, assignment of a participant identifier, and verbal permission to record. The overview below includes only the substantive interview domains relevant to the present analysis and the four themes reported in the Results. Questions and probes were used flexibly according to participants' responses. Topics outside the scope of this paper, as well as procedural instructions for interview setup, recording, field notes, transcript review, and file management, are not included.

Interview domain	Focus of questions and probes
Sexual health knowledge and informal learning	Formal and informal sexual health education; information learned from school, family, peers, social media, online sources, and personal experience; remaining knowledge gaps concerning STIs, condoms, testing, treatment, communication, and pregnancy prevention.
Sexual socialization, and peer influences	Expectations about masculinity and sexual experience; peer attitudes, social comparison, perceived pressure to be sexually active, and participants' responses to or resistance to these expectations.
Cultural, family, and religious influences	Family, cultural, and religious messages about sex, relationships, abstinence, responsibility, pregnancy, and readiness, and how these expectations shaped sexual and reproductive decisions.
Personal experiences and developing sexual autonomy	Personal and relational experiences that influenced sexual decision-making, including how participants developed individual values, boundaries, readiness, and independence from external expectations.
Perceptions of sexual risk and situational decision-making	How participants understood sexual risk and evaluated partners and situations, including partner familiarity, trust, relationship context, condom availability, and circumstances in which recognized risks were accepted.
STI prevention, testing, communication, and stigma	Condom use, testing, partner communication, disclosure, perceived STI status, stigma, experiences with diagnosis or treatment, and changes in behavior following STI-related experiences.
Reproductive responsibility and pregnancy prevention	Pregnancy prevention practices, perceived consequences of unintended pregnancy, shared responsibility with partners
Campus sexual health resources and support	Awareness, accessibility, affordability, visibility, and use of campus-based sexual health education, condoms, testing services, health centers, and related student support.
